# Development and validation of a multiplex fluorescent microsphere immunoassay assay for detection of porcine cytokines

**DOI:** 10.1016/j.mex.2019.05.013

**Published:** 2019-05-17

**Authors:** S.A. Hall, S.H. Ison, C. Owles, J. Coe, D.A. Sandercock, A.J. Zanella

**Affiliations:** a SRUC, West Mains Road, Edinburgh, United Kingdom; b World Animal Protection, United Kingdom; c University of Nottingham, School of Biosciences, United Kingdom; d Universidade de São Paulo, Faculdade de Medicina Veterinária e Zootecnia, Campus Pirassununga, Brazil

**Keywords:** Fluorescent microsphere immunoassay (FMIA), Porcine, Multiplex, Cytokine, FMIA, Brain, Placenta, Synovial tissue, Synovial fluid, Plasma, Validation

## Abstract

Cytokines are cell signalling proteins that mediate a number of different physiological responses. The accurate measurement of cytokine profiles is important for a variety of diagnostic and prognostic scenarios in relation to animal health and welfare. Simultaneous quantification of cytokine profiles in a single sample is now possible using fluorescent microsphere immunoassays (FMIA). We describe the development and validation of a novel multiplex assay using the Bio-Plex® 200 system to quantify cytokines in five different porcine tissues (brain, placenta, synovial tissue and fluid, plasma). The cytokine profiles are both tissue, and research hypothesis, -dependent but include Interleukin-1beta (IL-1β), Interleukin-4 (IL-4), Interleukin-6 (IL-6), Interleukin-8 (IL-8), Interleukin-10 (IL-10) and Tumor necrosis factor (TNF-α).

This methods paper is reported in two parts: the development of a FMIA for porcine tissues and validation of pre-treatment for optimal cytokine recovery in porcine brain, placenta, synovial tissue and plasma. Validation steps are critical in ensuring an assay is suitable for novel sample types.

This technique advances traditional ELISAs by:

• FMIA provides insight into the profiles of multiple porcine cytokines in certain situations (e.g. disease, parturition).
• Use of the Bio-Plex® 200 system to investigate novel sample types, including brain, placenta and synovial tissue.
• Multiplexing utilises a fraction of the sample volume compared with multiple ELISAs.

## Graphical abstract

**Figure:**
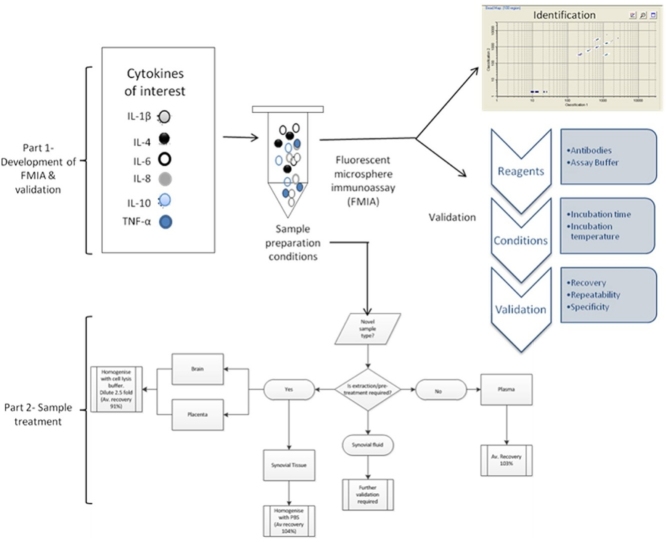

**Specifications Table**

**Table:** 

**Subject Area:**	*Agricultural and Biological Sciences*
**More specific subject area:**	*Animal health and biomarker detection*
**Method name:**	*Fluorescent Microsphere Immunoassay (FMIA)*
**Name and reference of original method:**	Bjerre,M., Hansen,T.K., Flyvbjerg,A. and Tonnesen,E., 2009. Simultaneous detection of porcine cytokines by multiplex analysis: Development of magnetic bioplex
**Resource availability:**	*Bio-Plex 200 Hardware: Bio-Rad – Bio-Rad, UK* *Software: Bio-Plex Manager v6.1 – Bio-Rad UK* *All reagents for Bio-Plex from Bio-Rad, UK* MagRack 6 (GE Healthcare) *Optional:* Bio-Plex Pro II Wash Station - Bio-Rad, UK *Antibodies – RnD systems (*https://www.rndsystems.com/*)* *Other chemicals and reagents – Sigma, ThermoFisher, Gibco*

## Method details

### Part 1 development of fluorescent microsphere immunoassay (FMIA)

#### Covalent coupling of capture antibodies to magnetic carboxylated microspheres

For each cytokine, the relevant capture antibody was coupled following manufacturer’s instructions, using reagents from the Bio-Plex amine coupling kit (Bio-Rad, UK) except where specified. All reagents were brought to room temperature (RT) before use. Briefly, 100 μl (1.25 × 10^6^ microspheres) of fluorescently distinct microspheres (beads) were separated from the supernatant using a magnetic separator and washed in 100 μl bead wash buffer by vortexing for 30 seconds (s). The supernatant was removed by magnetic separation again and then the washed beads were resuspended in 80 μl of bead activation buffer by vortexing for 30 s. To this, 10 μl of S-NHS (50 mg/ml; Fisher, UK) and 10 μl of EDC (50 mg/ml; Fisher, UK) prepared in bead activation buffer were added and mixed gently by vortexing. The tube was incubated in the dark with shaking for 20 min at room temperature (RT). After incubation, 100 μl phosphate buffered saline (PBS; pH 7.4) was added and vortexed for 10 s. The supernatant was removed by magnetic separation and the PBS wash step repeated. The beads were then resuspended in 100 μl PBS and vortexed for 30 s. The recombinant cytokine protein was added to the activated beads and mixed by vortexing and then incubated for 2 h in the dark at RT with shaking.

The supernatant was removed by magnetic separation, using a MagRack 6 (GE Healthcare) and coupled beads were resuspended in 500 μl PBS. The supernatant was again removed, and beads were resuspended in 250 μl of blocking buffer, vortexed for 15 s. and incubated in the dark at RT for 30 min with shaking. The supernatant was removed by magnetic separation and beads were resuspended in 500 μl of storage buffer. Further concentration of the beads was obtained when the supernatant was removed by magnetic separation and then coupled beads were resuspended in 150 μl of storage buffer. The bead concentration was determined using an automated cell counter (TC10 Bio-Rad, UK). The coupled beads were stored in the dark at 2–8 °C until use.

#### Singleplex and multiplex procedures

Capture and detection antibodies and recombinant cytokines were purchased as matched pairs if available which were commercially validated for binding to different epitopes on the antigen (Table 1). This increased the likelihood of antibody reactivity for each cytokine and decreased likelihood of cross-reactivity with other cytokines. Individual cytokine measurements were initially optimised as a singleplex for IL-1β, IL-4 IL-6, IL-8, IL-10, TNF-α) before combining them to perform a multiplex, which was then further optimised. Optimisations included the amount of capture antibody coupled to magnetic microspheres, detection antibody concentration, incubation time and assay buffer matrix. Optimal antibody (capture and detection) concentrations are summarised in Table 1. The optimal assay buffer used was 81% distilled water, 10% Reagent diluent (R&D systems, UK) and 9% heat inactivated foetal calf serum (Gibco, UK). For the FMIA, 50μl of magnetic microspheres coupled with 20 μg/ml of capture antibody were first added to a 96 well, black, flat-bottomed plate and washed twice with wash buffer using a Bio-Plex Pro II Wash Station (Bio-Rad, UK). To this, 50 μl of prepared recombinant standards, unknown samples, controls or blanks (assay buffer only) were added. The plate was then incubated in the dark with shaking at RT for 2 h. To reduce bubbles from forming (and beads from sticking to sides) during incubation a high shaking setting (900–1100 rpm) was used for first 30 s then lower (300 rpm) for the rest of the incubation time. The plate was washed three times as before, then 100 μl of the detection antibody (in assay buffer) was added to the wells and the plate was incubated as before for 40 min. The plate was washed three times as previously, then 50 μl of diluted Streptavidin-PE solution (Bio-Rad) was added and the plate was incubated for a further 20 min. The plate was washed for a final three times, and then 125 μl of assay buffer was added. The plate was incubated with shaking for 5 min then the reaction was measured using a Bio-Plex 200® instrument and analysed with the Bio-Plex Manager software version 6.1. For details of measurements taken during the run see Hall et al. [1]. The instrument was calibrated daily and validated monthly using bead standards (Bio-Rad). Mean Fluorescent Intensity (MFI) for 100 microspheres corresponding to each individual cytokine analyte was recorded for each well. All reported MFI measurements were background corrected by the software (normalised) (F − F_o_), where F_o_ was the background signal determined from the fluorescence measurement of the blank. The standard curve was produced using logistic 5PL regression where recovery was in the 70–130% range as previously deemed acceptable [2].

**Table 1:** Antibodies (with optimised concentrations), standards and beads used for each cytokine in the multiplex assay.

Analyte	Reagent	Catalogue #	Description/bead region	Optimised antibody concentration (μg/ml)/bead region	Source
IL-1β	Capture Ab	MAB6811	Mouse anti-porcine	20	R&D systems
	Detection Ab	BAF681	Biotinylated goat anti-porcine	0.5	R&D systems
	Standard	681-PI	Recombinant Porcine IL-1 beta	–	R&D systems
	Bead	MC10029	Pro Magnetic COOH Beads 29	29	Bio-Rad
IL-4	Capture Ab	AF654 BAF654	Goat anti-porcine	20	R&D systems
	Detection Ab	654-P4	Biotinylated goat anti-porcine	0.75	R&D systems
	Standard	MC10064	Recombinant Porcine IL-4	–	R&D systems
	Bead		Pro Magnetic COOH Beads 64	64	Bio-Rad
IL-6	Capture Ab	AF686 BAF686	Goat anti-porcine	20	R&D systems
	Detection Ab	686-PI	Biotinylated goat anti-porcine	0.5	R&D systems
	Standard	MC10026	Recombinant Porcine IL-6	–	R&D systems
	Bead		Pro Magnetic COOH Beads 26	26	Bio-Rad
IL-8	Capture Ab	MAB5351 BAF535	Mouse anti-porcine	20	R&D systems
	Detection Ab	535-IN	Biotinylated goat anti-porcine	0.5	R&D systems
	Standard	MC10045	Recombinant Porcine IL-8	–	R&D systems
	Bead		Pro Magnetic COOH Beads 45	45	Bio-Rad
IL-10	Capture Ab	MAB693 BAF693	Mouse anti-porcine	20	R&D systems
	Detection Ab	693-PI	Biotinylated goat anti-porcine	0.5	R&D systems
	Standard	MC10035	Recombinant Porcine IL-10	–	R&D systems
	Bead		Pro Magnetic COOH Beads 35	35	Bio-Rad
TNF-α	Capture Ab	MAB690 BAF690	Mouse anti-porcine	20	R&D systems
	Detection Ab	690-PT	Biotinylated goat anti-porcine	0.5	R&D systems
	Standard	MC10055	Recombinant Porcine TNF-α	–	R&D systems
	Bead		Pro Magnetic COOH Beads 55	55	Bio-Rad

Ab: antibody, Source: R&D Systems, (Abingdon, UK), Bio-Rad, (Hemel Hempstead, UK).

#### Validation of FMIA

Each cytokine (singleplex) was optimised individually and validated as below before an additional cytokine was added sequentially form a multiplex. The FMIA assays were validated using a number of measures.

Assay range and sensitivity: These were the concentrations as measured and reported by the Bio-Plex Manager software on completion of the assay run.

Recovery: For each cytokine standard the recovery of known amounts of cytokines (range 10,000–40.06 pg/ml) had to be within the 70–130% range to be included as successful detection [2]. In addition, samples were spiked with known amounts of cytokine to calculate spiked recovery. This also had to be within the 70–130% range.

Repeatability: To verify the repeatability of the assay, dilutions of recombinant cytokine standards were prepared for each cytokine and used in triplicate in the FMIA over a 3-day period of time [3] and expressed as inter and intra-assay variation. The determination of intra-assay repeatability was evaluated by analysing multiple replicates (n = 3) of seven recombinant cytokine standards with known concentrations during a single assay run and expressed as the coefficient of variation (CV) of repeated measurements. Inter-assay variability was studied using seven different concentrations of standards, analysed in triplicate over three different days and expressed as the CV of repeated measurements [3].

Cross-reactivity (specificity): A multiplex FMIA was set up with standards containing all capture and detection antibodies and all recombinant cytokines. Unknown wells contained all capture antibodies and cytokine recombinants but only one detection antibody to test specificity for each matched pair of antibodies. Cross reactivity was defined as the percentage of nonspecific, cross reacting signal detected relative to the specific signal for that analyte.

### Part 2 extraction of cytokines from porcine tissues

#### Brain tissue

Porcine brain samples were collected as part of a previous study and stored at −80 °C [4] and prepared using a modification of procedures described by Hulse et al. [4]. The hippocampus from the left hemisphere was removed from a brain slice of each pig (n = 14), weighed, crushed to a powder with liquid nitrogen and transferred to a 1.5 ml microtube. To this, 500 μl of cell lysis buffer (Bio-Rad, UK) with 1 μl stock solution was added and homogenised with a hand-held homogeniser (VWR VDI12). The total protein content was measured using Quick Start Bradford Protein Assay (Bio-Rad, UK) according to manufacturer’s instructions (Bio-Rad Quick start Bradford Assay) [5]. Brain extracts were frozen in aliquots (individually and equally pooled) at −80 °C pending FMIA analysis. Pooled extracts were spiked (with 4000 pg/ml) with both individual cytokines and a mixture of all six cytokines and then also diluted 2.5 fold, to validate the assay, ensuring recovery was always within 70–130% range.

#### Placenta

Pig placenta tissue was collected as part of a previous study and stored at −80 °C [6] and was extracted by the same method as the brain tissue above. For both tissues types, before analysis samples were pooled and both pooled and pooled-spiked (with 4000 pg/ml cytokines) samples were serially diluted six times (2.5 fold dilutions) and percentage recoveries were calculated for all dilutions.

#### Synovial tissue

Synovial tissue (ST) was collected from a pig model of spontaneous joint disease and lameness where a cytokine response was being investigated [7]. Synovial samples were collected from the knee joints of ‘Landrace × Duroc × Large White’ female pigs of different ages. Following humane euthanasia the knee joint was carefully opened and aseptic aspiration was used to obtain a sample of synovial fluid, before a lateral or frontal joint capsule sample was excised and the surface of the articular cartilage examined. A sample of joint synovial tissue was taken at the site of excision. Synovial tissue samples (approx. 1 g) and synovial fluid were rapidly frozen on dry ice in DNase/RNase-free 2 ml cryovials and stored at −80 °C pending analysis.

Three different extraction buffers were tested: (1) radio-immunoprecipitation buffer (RIPA) lysis buffer, (2) cell lysis buffer and (3) phosphate-buffered saline (PBS) for the recovery and quantification of three cytokines (IL-1β, IL-6 and TNFα) using the FMIA.

For RIPA buffer extraction, approximately 0.3 g of synovial tissue was homogenised on ice in RIPA lysis buffer (3 ml) (Alpha Diagnostics #RIPA-50 lysis buffer kit for mammalian cell lysis buffer with protease phosphatase inhibitors cocktail) and left to fully solubilize the tissue proteins for 2 h on ice (following manufacturer’s guidelines). Synovial tissue lysates and synovial fluid supernatants were obtained following centrifugation of the homogenates at 3000 × *g* for 20 min at 4 °C. All lysates and supernatants were carefully pipetted off and transferred to new cryovial tubes and stored at −80 °C pending immunoassay analysis.

Cell lysis buffer extraction involved [5] further synovial tissue samples homogenised in lysis buffer with protease inhibitors. Protease Inhibitor Cocktail lyophilised powder (Sigma-Aldrich, catalogue # P2714) was reconstituted with Tissue Extraction Reagent I (lysis buffer) (Invitrogen, catalogue # FNN0071) just before use. After reconstitution, the solution is stable for 24 h at 4 °C, therefore aliquots were made for immediate use and freezing. 0.1 g of tissue was weighed per sample and homogenised as in the above procedure in a final volume of 1 ml buffer. Homogenates were centrifuged at 5000 × *g* for 5 min (4 °C), according to the manufacturer’s instructions, and aliquoted and frozen as above.

For PBS cytokine extraction, approximately 200 mg of synovial tissue was added to 2 ml x1 PBS and homogenised over ice using a hand-held homogeniser (VWR VDI12). Samples then underwent two freeze-thaw cycles before centrifuging at 5000 × *g* for 5 min. Supernatants were collected, aliquoted and immediately frozen. Some samples required a second centrifugation due to fat globules remaining in the supernatant. We tested recovery of spiked samples for each extraction method (RIPA, cell lysis, PBS) by spiking with a known concentration of cytokine standards (4000 pg/ml) and RIPA buffer extracted samples were also diluted 1:2, 4 and 8 to test for dilution effects.

Protein concentration of the all lysates was measured using a Bradford assay according to manufacturer’s instructions (Bio-Rad Quick start Bradford Assay) as before.

#### Synovial fluid

Synovial fluid (SF) is a non-newtonian fluid with a complex matrix including hyaluronic acid, lubricin, proteinases, and collagenases. It may also contain rheumatoid factor (RF) and heterophilic antibodies [8,9]. SF was collected at the same time as the synovial tissue (see above). For validation experiments, a pool of SF from four pig samples was prepared. It was centrifuged at 1000 × *g* for 15 min. and supernatant used.

For the recovery and quantification of three cytokines (IL-1β, IL-6 and TNFα) using the FMIA, we tested recovery of spiked samples following dilution (neat, 1:2, 4, 8), hyaluronidase treatment, centrifugal concentration using vivaspin filter columns and incubation with a blocking buffer.

For hyaluronidase treatment we added 2 mg/ml hyaluronidase (sigma catalogue H3506) in 0.02 M PBS to the SF and incubated for 1 h at RT [10].

In an attempt to filter out the larger molecules such as hyaluronan, chondroitin sulphate and keratan sulphate [10] from the SF we used vivaspin columns (Sartorius vivaspin 500 columns, 50KMWCO, PES membrane). SF (was loaded onto the filter and centrifuged at 14,000 × *g* for 9 min. Samples were spiked before (pre) and after (post) filtering with the columns with a known concentration of cytokine standards (4000 pg/ml). They were also diluted by 1: 2, 5, 10, 20, 40 to ascertain any dilution effects.

In an attempt to block antibodies and RF interference which can cause capture and detection antibodies to crosslink [8,11], a pooled SF sample was also incubated with a blocking buffer of either bovine plasma (Biosera) or a 1:1 equine and caprine serum (Sigma-Aldrich). Synovial fluid was incubated (1:1) for 30 min at RT before centrifuging at 1000 × *g* for 15 min.

#### Blood plasma

Blood was collected from pigs post-farrowing from tail or ear veins, as part of a separate study investigating post-partum biomarkers of inflammation [12].

A 6 ml EDTA vacutainer (Henry Schein Animal Health, Dumfries, Dumfries and Galloway, UK) was used to collect a blood sample. For more detailed methodology see Ison et al. [12]. Blood was immediately placed on ice, then moved straight into a refrigerated centrifuge (at 4 °C) and centrifuged for 15 min at 1400 × *g*. Plasma was pipetted into four 1.5 ml pre-labelled tubes and frozen at −80 °C to be assayed at a later date. Samples were removed from the −80 °C freezer on the morning of the assay, put on ice to defrost then three pooled samples were created. These were analysed for three cytokines (IL-1β, IL-6, TNF-α) neat and diluted 1:2 following previous results with plasma [2]. In addition, we analysed the pooled samples spiked with known concentrations of cytokines at high (4000 pg/ml) and low (640 pg/ml) and diluted 1:2 for spiked recovery calculations.

## Method validation results

### Part 1 development of multiplex assay

Singleplex FMIA were developed for IL-1β, IL-4, IL-6, Il-8, IL-10 and TNFα which were then combined into a 6-plex. The selection of cytokines was based on their role as welfare and pain biomarkers [13]. These cytokines were chosen to produce a broad profile of cytokines with both pro- and anti-inflammatory roles, but were also constrained by commercial availability of porcine antibodies.

**Fig. 1:**
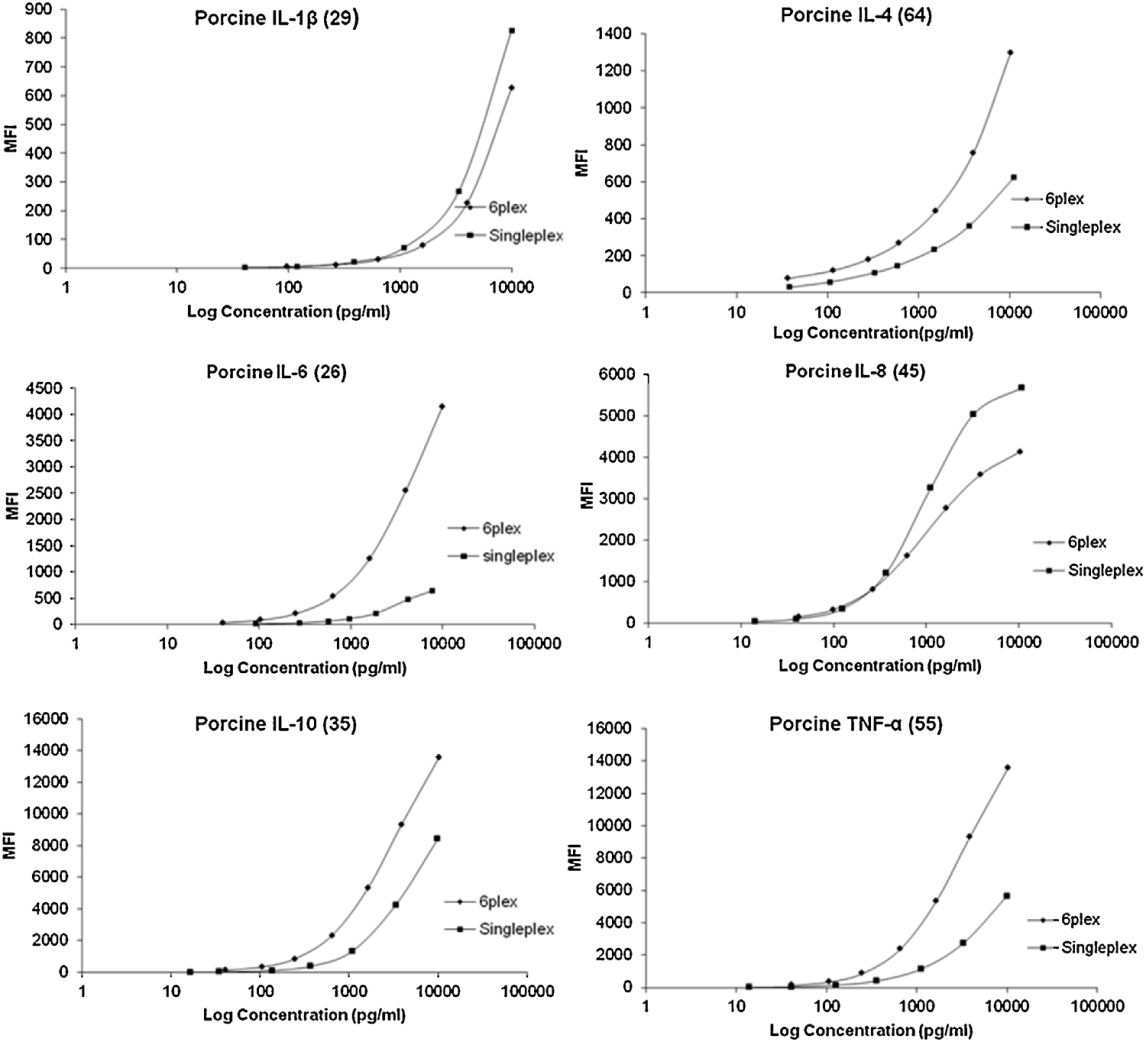
Standard curves for singleplex (squares) and 6-plex (diamonds) for each of the six cytokines IL-1β, IL-4, IL-6, Il-8, IL-10 and TNFα shown by mean fluorescent intensity (MFI) for seven serially diluted recombinant cytokine standards. Numbers in parantheses are Luminex microsphere (bead) numbers used.

Standard curves (MFI versus concentration) following a 2.5 fold dilution for each cytokine followed a similar trend for both singleplex and 6-plex however fluorescence was higher in 6-plex compared to singleplex for IL-4, IL-6, IL-10 and TNFα, but the converse was seen for IL-1β and IL-8 (Fig. 1). This may indicate a change in sensitivity, which may be due to the increase in microspheres present. MFI values were very different for each cytokine over the standard range, with maximum MFI ranging from 800 to 14,000 (Fig. 1).

The 6-plex had a range over 4 logs from 10,000 to 40.96 pg/ml for each cytokine except IL-1β (lower 102.40 pg/ml) which is comparable to previous assays [2] and covers the range of cytokines detected in porcine serum [3]. Inter and intra-assay variation was low for all cytokines (<7%), which was an improvement over previous assays (3–18%) [2,3] (Table 2).

**Table 2:** Validation of porcine six-plex assay. Assay range of detection, sensitivity, intra- and inter-assay variation and assay specificity (% cross reactivity).

Target cytokine (bead region)	Assay range (pg/ml)		Sensitivity (pg/ml)	Intra-assay (%CV)	Inter-assay (%CV)	Cross reactivity (%)					
	min	max				IL-1β	IL-4	IL-6	IL-8	IL-10	TNF-α
IL-1β (29)	102.40	10,000	97.55	3.72	3.75	107.96	0.50	0.50	0.21	0.35	0.00
IL-4 (64)	40.96	10,000	36.07	5.10	5.25	0.50	98.10	0.80	0.50	0.60	0.60
IL-6 (26)	40.96	10,000	40.53	3.73	4.14	1.46	0.83	110.29	0.59	0.39	1.40
IL-8 (45)	40.96	10,000	42.05	4.81	5.65	1.42	0.51	0.49	97.51	0.34	0.51
IL-10 (35)	40.96	10,000	40.58	4.10	4.15	1.40	0.62	0.45	0.18	108.07	0.26
TNF-α (55)	40.96	10,000	38.64	4.64	4.95	1.21	0.62	0.44	0.18	0.31	101.96

#### Cross reactivity

Singleplex reactions for each of the six cytokines were checked systematically against the other cytokine reactions to ensure they were specific. No cross reactivity was observed for any of the 6-plex cytokine reactions (Table 2), which ensures that there will be no false positive results when samples are analysed with the multiplex.

### Part 2 detection of cytokines in porcine tissue

#### Brain

A mean of 9.36 mg/ml (±0.91) of protein was extracted from brain tissue using the cell lysis method. Three cytokines were detected in the brain samples, with spiked recovery within the 70–130% range, with average concentrations of pooled samples: IL-1β (301.98 pg/ml CV% 2.32%), IL-6 (16.03 pg/ml, CV% 3.65) and IL-10 (9.65 pg/ml CV% 10.88).The levels of other cytokines (IL-4, IL-8 and TNF-α) were all below the level of detection of the FMIA. Dilution of spiked pooled sample by 1:2.5 gave the optimum percentage recovery, with an average of 91% (±s.e.m 13.3).

#### Placenta

A mean of 6.40 mg/ml (±0.63) of protein was extracted from placenta using the cell lysis method. Porcine placenta cytokine levels were assessed using the 6-plex. Initially, placenta and spiked samples of ‘normal’ and ‘small’ placenta were run at four dilutions (2.5 fold serial dilutions). A dilution of 1:2.5 gave the optimum percentage recovery across all cytokines, with an average of 117% recovery (±s.e.m 24.0). Three cytokines were present in measurable amounts in placental samples IL-1β (median 265.63 pg/ml, range 187.05–344.2), IL-6 (4169.41 pg/ml range 3223.57–4169.41), IL-8 (258.26 pg/ml, range 147.58–368.94). The other three cytokines (IL-4, IL-10 and TNF-α) were below the level of detection (see Table 2 for minimum range for each cytokine) in these particular placenta samples. There were no Bio-Plex sample errors, such as low bead number for either placenta or brain tissue (data not shown).

#### Synovial tissue lysate

Of the three pre-treatment extraction methods used to extract cytokines from synovial tissue, (RIPA, cell lysis buffer and PBS) there was a range of recoveries observed (Table 3). The extraction method appeared to differentially affect the recoveries of each of the three cytokines tested (IL-1β, IL-6 and TNFα) with IL-6 consistently showing the highest recovery, emphasising the importance of validating all analytes in an assay. The highest recoveries were seen with PBS buffer (IL-1β 89%, IL-6 130% and TNFα 94%), so this method was chosen for subsequent analyses.

**Table 3:** Synovial tissue comparison of spiked recoveries (with 4000 pg/ml) for extraction with RIPA buffer (diluted 2, 4, 8), cell lysis buffer and PBS. Italicised numbers represents unacceptable recoveries (out with 70–130%).

Synovial Tissue	RIPA buffer			Cell lysis buffer	PBS extraction
Cytokine	Dilution 1:2 spiked sample % recovery	Dilution 1:4 spiked sample % recovery	Dilution 1:8 spiked sample % recovery	Spiked sample % recovery	Spiked sample % recovery
IL-1β	*49*	*57*	*62*	*49*	89
IL-6	96	89	86	*44*	130
TNF-α	*16*	75	84	*13*	94

#### Synovial fluid

Four pre-treatments were tested with SF (dilution in assay buffer, hyaluronidase treatment, viva spin columns and incubation with serum). During the process of assay validation, it was discovered that the recovery of known concentrations of cytokines in spiked samples of SF was very variable and consistently <50% (range 11–48% with optimal dilutions for all cytokines). SF was initially diluted to reduce the fluid’s viscosity, and potential interference from antibody/rheumatoid factors present, however for the three cytokines tested (IL-1β, IL-6 and TNFα) the spiked recoveries ranged from 7 to 48%. Comparison of incubating with and without hyaluronidase, only increased recovery from 8 to 23% (low spike) and 22 to 29% high spike. Spin column recoveries were also inadequate as columns appeared to filter out cytokines as well as molecules that were interfering with the assay, with spiked recoveries only improving if cytokine was added after the column filtration. Incubation of SF with bovine plasma improved spike detection by 10–55% and incubation with horse/goat serum improved spike detection by 14–45%. To date, none of these approaches has yielded significantly improved analyte recovery or identified the most effective pre-treatment for SF. Further work on the SF samples is on-going to resolve analyte recovery, and reliability and repeatability of measurement. Detail of specific recoveries for each test in Supplementary material (Tables 5–8).

#### Blood plasma

Plasma was chosen for validation rather than serum due to previous inconsistencies observed while validating cytokines in horse blood [1]. All three cytokines (IL-1β, IL-6, TNF-α) were detected in neat pooled samples but diluting 1:2 resulted in levels close to the lowest level of detection and unacceptable percentage recovery values for TNF-α (Table 4). Moreover, pooled plasma spiked with known cytokines resulted in acceptable recoveries if run neat however resulted in poor recovery values when diluted 1:2 (Table 4). Therefore, porcine plasma samples were analysed neat for all further plasma cytokine measurements in pigs.

**Table 4:** Average percentage recoveries for pooled samples of plasma (diluted 1:2) and spiked with high (4000 pg/ml) and low (640 pg/ml) cytokines. Italicised numbers represents unacceptable recoveries (out with 70–130%).

		Average % recovery	Average % recovery	Average % recovery
	Dilution	IL-1β	IL-6	TNF-α
Spiked plasma HIGH (4000 pg/ml)	1	102	100	106
Spiked plasma LOW (640 pg/ml)	1	95	101	111
Spiked plasma HIGH (4000 pg/ml)	2	105	125	*135*
Spiked plasma LOW (640 pg/ml)	2	109	*168*	*219*
Pooled samples	2	106	97	*66*

## Conclusion

The development of this robust yet flexible FMIA assay, which has improved sensitivity over previous assays, is capable of measuring multiple cytokines simultaneously in porcine biological samples. Wyns et al. [14] reported that bead-based assays are more efficient at capturing soluble antigens than surface-coated ELISAs. Overall, our standard curve range and assay sensitivity is similar to previous FMIA and covers a wider range than many commercial ELISA kits [2,15] and we have shown its use with a wider range of sample types. The standard curve range of IL-1β, in this multiplex has a reduced sensitivity compared to the other analytes, however this has been observed previously [3], which allows us to measure porcine plasma without need for dilution. This assay will reduce sample volume, time and cost associated with single ELISAs yet also providing a more comprehensive picture of the suite of cytokines produced in different tissues, allowing investigation into immunological response to specific challenges. The added value of this tool is its potential, following appropriate validation steps, to have additional cytokines of interest added. The panel of cytokines chosen for FMIA should be biologically relevant to the research question being asked. This porcine assay, validated for use in brain, placenta, synovial tissue lysates and plasma adds to the laboratory toolbox for this increasingly utilised animal model for biomedical science research.

The extraction part of this assay development has highlighted how caution should be taken when working with previously untested biological samples, such as synovial fluid and the importance of validation of any novel assay. We did not observe the same recovery values as [10] for treated SF, therefore we do not use this assay for this sample type at present. In our attempts to measure cytokines in synovial fluid we did an extensive literature review to better understand any sample pre-treatment required. In the majority of published reports the analyte recovery data was often lacking or not discussed and this highlights the importance of internal validation in order to obtain reliable and consistent results.

This technical note describes a FMIA protocol for measurement of multiple cytokines in porcine samples and the different pre-FMIA extraction methods required for optimal analyte recovery and quantification.
