# Supplementary material for: Development and validation of a multiplex fluorescent microsphere immunoassay assay for detection of porcine cytokines
Source: MethodsX. 2019 May 17;6:1218–27. doi: 10.1016/j.mex.2019.05.013 (PMC6545349; doi:10.1016/j.mex.2019.05.013)
Supplement: Supplementary file 1 [file mmc1.docx]

**Supplementary material *and/or* Additional information:**

**Information relating to synovial fluid pre-treatment results**

**Table 5. Recoveries of Spiked SF samples following dilution.** Italicised numbers represents unacceptable recoveries (out with 70-130%).

|  | **IL-1β** | | **IL-6** | | **TNF-α** | |
| --- | --- | --- | --- | --- | --- | --- |
| **Dilution** | Average recovery % | CV% | Average recovery % | CV% | Average recovery % | CV% |
| 2 | *48* | 12 | *25* | 6 | *13* | 7 |
| 4 | *35* | 13 | *27* | 5 | *7* | 20 |
| 8 | *43* | 7 | *40* | 7 | *13* | 24 |

**Table 6. Synovial fluid following hyaluronidase (HYAL) treatment**

|  |  | **without HYAL** | | **with HYAL** | |
| --- | --- | --- | --- | --- | --- |
| Treatment | Spike conc (pg/ml) | average % | CV% | average % | CV% |
| BUFFER spike High | 5000 | 116 | *2* | *21* | *3* |
| BUFFER Spike Low | 500 | 138 | *2* | *19* | *2* |
| SF spike High | 5000 | *22* | *7* | *29* | *1* |
| SF Spike Low | 500 | *8* | *9* | *23* | *3* |

**Table 7. SF recoveries after vivaspin columns (spiked pre/post spin).** Italicised numbers represents unacceptable recoveries (out with 70-130%).

|  |  | % Recovery at Dilution | | | | |
| --- | --- | --- | --- | --- | --- | --- |
| Cytokine | Spiked | 2 | 5 | 10 | 20 | 40 |
| **IL-1β** | **PRE spin** | *1* | *1* | ***** | *1* | ***** |
|  | **POST spin** | 102 | 76 | *69* | *69* | 70 |
| **IL-6** | **PRE spin** | 0 | 1 | 1 | 1 | 2 |
|  | **POST spin** | *67* | *68* | 87 | 72 | 82 |
| **TNF-α** | **PRE spin** | 0 | *** | 2 | 0 | *** |
|  | **POST spin** | 124 | 79 | 87 | *69* | 82 |

**Table 8. Synovial Fluid recoveries after incubation with blocking buffers.**

|  | Synovial Fluid % Recovery with Blocking Buffer Incubations | | | |
| --- | --- | --- | --- | --- |
|  | Bovine Plasma | | Horse/Goat Serum | |
| Cytokine | No Incubation | Incubated | No Incubation | Incubated |
| **IL-1β** | *5* | *60* | *29* | *64* |
| **IL-6** | *13* | *22* | *4* | *18* |
| **TNF-α** | *1* | *1* | *0* | *45* |
